# Determination of Total Chlorogenic Acids in Commercial Green Coffee Extracts

**DOI:** 10.1089/jmf.2018.0039

**Published:** 2019-03-15

**Authors:** Joe A. Vinson, Xi Chen, Deanne Dulik Garver

**Affiliations:** ^1^ Department of Chemistry, Loyola Science Center, University of Scranton, Scranton, Pennsylvania.; ^2^ Department of Science, Mathematics and Computer Science, Marywood University, Scranton, Pennsylvania.

**Keywords:** *caffeine*, *chlorogenic acids*, *green coffee extract*, *HPLC*, *LC/MS*

## Abstract

Obesity and type II diabetes are serious health problems and are among the leading causes of death. There are a few prescription weight loss drugs, but they have a high cost and their adverse effects have limited their widespread use. For the consumer, the use of dietary supplements represents a natural and presumably safer means of losing weight. A high-pressure liquid chromatography (HPLC) method was developed to provide a simple, inexpensive method for analysis of 54 commercially available extracts of green coffee beans. Both chlorogenic acids (CGAs), which are the purported bioactives, and caffeine were measured using 5-chloroquinic acid as the standard and published extinction coefficients for the other monomeric and dimeric CGAs present. The average labeled dose of CGA was 233 mg, whereas the average calculated by HPLC analysis was only 157 mg. Thus, the consumer is likely to obtain product containing a little more than half of the reported label amount of CGA. Caffeine levels ranged from 0% to 17%. The marketing literature touts 50% CGA content as being the gold standard of green coffee bean extract products. Based on this value, only 28% of the commercial products we studied met this goal.

## Introduction

The most recent government statistics state that in 2013–2014, 70.7% of Americans over the age of 20 were overweight, including the classification as obese.^1^ A recent report in Packaged Facts^2^ stated that 28% of American adults (66 million) are trying to lose weight and a further 13% (32 million) are trying to maintain their weight. According to a recent study, since 1980 worldwide, the proportion of women who are overweight or obese has gone from 30% to 38%; men have risen from 29% to 40%, thus overtaking women in percentage overweight or obese.^3^ There are now five Food and Drug Administration-approved weight loss pharmaceuticals that have been approved in the past several years. There are also numerous natural products that purport to cause weight loss available over the counter. In addition, because of Dr. Oz's television show in 2012 touting of green coffee bean extract as a miracle weight loss product, a large number of green coffee extracts (GCEs) became commercially available. Amazon currently lists >10,000 commercial sources of GCE on their website (accessed September 2018). There have been a few clinical studies of GCE from the United Kingdom, Norway, and France and three of these studies were subjected to a meta-analysis.^4^ This report states that there was an average of 5 pounds lost among study participants, but the authors concluded that “more rigorous trials are needed as the studies had a small number of participants and were short-term.” There is a weight loss book with green coffee bean in the title^5^ in which GCE is incorporated in a healthy diet. A combination of GCE, green tea extract, *Garcinia cambogia* (Malabar tamarind), and *Lagerstroemia speciosa* (banaba) in 12 weeks produced a weight loss of 5 pounds versus 1 pound for the placebo.^6^ GCE dosage in the combination product was 120 mg per day.

Besides weight loss, GCE and green coffee have been used for other medical purposes. A GCE capsule of 400 mg dosage was given twice a day for 8 weeks versus a placebo to subjects with metabolic syndrome. This is a cluster of conditions, including increased blood pressure, high blood sugar, body fat around the waist, and abnormal cholesterol or triglyceride levels. The GCE regimen caused a significant drop in systolic blood pressure, glucose waist circumference, insulin resistance, and appetite score. Weight and body mass index (BMI) were also significantly decreased.^7^ The most recent study published in this journal^8^ investigated GCE at a dose of 400 mg three times per day for 12 weeks to 15 subjects with impaired glucose tolerance (prediabetic). There were significant decreases in fasting blood glucose, insulin resistance, body weight (5.6 lbs lost), BMI, waist circumference, triglycerides, cholesterol, and low-density lipoprotein cholesterol.

**FIG. 1.**
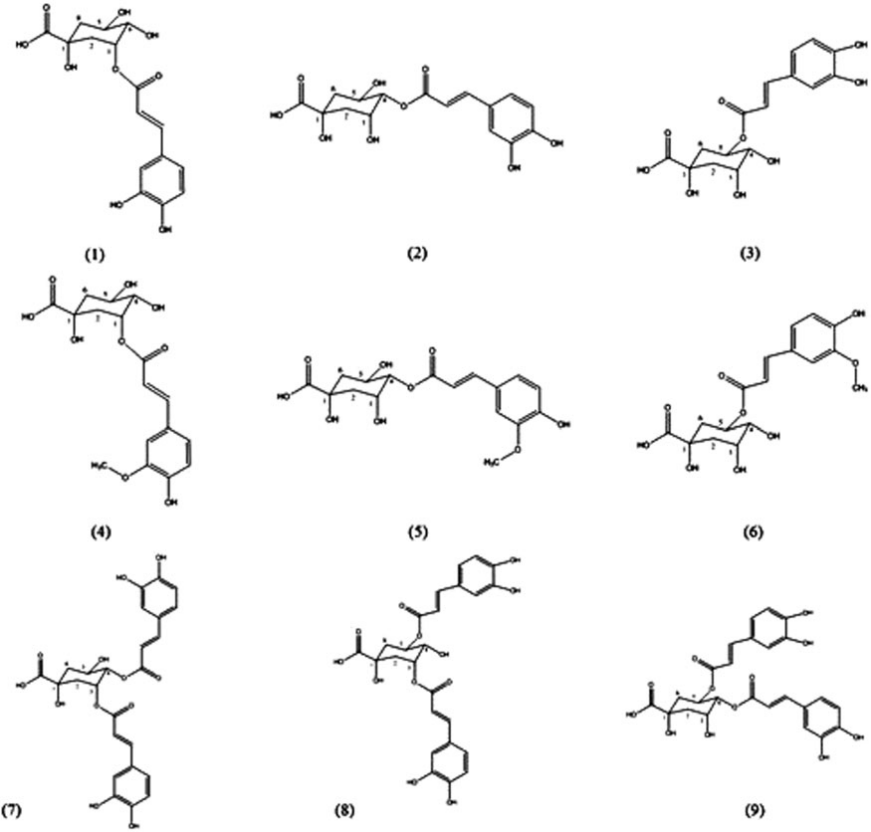
Monomers and dimers of CGAs found in green coffee: (1) 3-O-caffeoylquinic acid, (2) 4-O-caffeoylquinic acid, (3) 5-O-caffeoylquinic acid, (4) 3-O-feruloylquinic acid, (5) 4-O-feruloylquinic acid, (6) 5-O-feruloylquinic acid, (7) 3,4-di-O-caffeoylquinic acid, (8) 3,5-di-O-caffeoylquinic acid, and (9) 4,5-di-O-caffeoylquinic acid. CGAs, chlorogenic acids.

Green coffee beans contain 5–14% of the reported major components, chlorogenic acids (CGAs),^9^ and these compounds are significantly reduced after roasting.^10^ The lower range is provided by extracts of *Coffea arabica* and the higher range by *Coffea canephora*. The GCE are concentrated extracts of unroasted green coffee beans. The roasting process destroys CGAs; therefore, the unroasted beans are used as the source for this nutritional supplement. Some subjects report excellent weight loss using GCE products and other results are unimpressive. One of the possible variables in the use of these products is the dosage of the active ingredient(s), which is not controlled, as are prescription weight loss drugs. GCE is a complex mixture primarily consisting of nine CGAs present as monomers and dimers (includes both caffeoylquinic and feruloylquinic acids as shown in Fig. 1). The most abundant component of CGA is 5-O-caffeoylquinic acid (IUPAC nomenclature) often misnamed in the literature as CGA^11^ although other constitutional isomers are also present in the mixture. Only the monomers and dimers present in GCE are bioavailable in humans.^12^

Thus, due to the increasingly researched medical uses and importance to the consumer, we decided to analyze GCE products from commercial sources available in the United States for both CGAs and the stimulant caffeine, which is also present in the extracts.

## Materials and Methods

### Solvents and analytical standards

Three commercial standards of 5-chloroquinic acid (5-CQA) were evaluated for purity: Sigma-Aldrich (USA), Chromadex (USA), and Phytolab (Germany). Caffeine standard was purchased from Sigma. Methanol and acetonitrile were high-pressure liquid chromatography (HPLC) grade and obtained from Fisher Chemical. Formic acid was reagent grade (Sigma) and was mixed at the appropriate concentration in water. Water was purified from a Millipore Direct Q UV3 system.

### Sample preparation and HPLC analysis

Fifty-four samples containing GCE were obtained from commercial sources from the Internet and at least two bottles of the same lot were opened. The samples consisted of capsules, soft gels, or packs of powdered extracts. Listed ingredients included excipients such as cellulose, silica, magnesium stearate, gelatin, rice bran, malt dextrin, titanium dioxide, and calcium carbonate. Other ingredients included black pepper, turmeric, raspberry ketone, mango, acai, kelp, grapefruit, apple cider vinegar, green tea, ginseng, chromium dinicotinate, and yerba mate. Samples were ground with a mortar or pestle to a powder, if necessary, and then were pooled. Aliquots of 30–50 mg were weighed on an analytical balance and extracted with 100 mL of methanol on a wrist shaker for 5 min and then centrifuged for 10 min. Aliquots of the solutions were then stored at −80°C until assayed.

The HPLC system utilized a Shimadzu LC-20AD with photodiode array (PDA) detection (Shimadzu SPD-M20A) at 325 nm for CGAs and caffeine at 275 nm. Chromatographic separation was achieved with an Agilent Zorbax RX C-18 column (4.6 mm × 250 mm) with a pore size of 5 *μ*m. The 5-CQA standard was analyzed with a 12.5-min gradient using the following conditions: (A) 0.1% formic acid in water; (B) 0.1% formic acid in acetonitrile; gradient elution, time 0 at 5% B to 12.5 min at 10% B then hold for 10 min at a flow rate of 1 mL/min. The retention time of the 5-CQA standard was 18 min.

CGA and related compounds were first identified by the order of elution^13^ and later by liquid chromatography (LC)/mass spectrometry (MS). All CGA had a similar ultraviolet (UV)-visible spectrum by PDA detection. For the HPLC/UV studies, the samples were analyzed using a batch analysis program at a flow rate of 1 mL/min using 0.1% formic acid in water (A) and 0.1% formic acid in acetonitrile (B) with gradient elution as follows: 0 min (5% B), 12.5 min (10% B), 22.5 min (10% B), and 60 min (30% B).

### Structural confirmation by LC-electrospray ionization/MS

LC-electrospray ionization/MS analysis was carried out using a Shimadzu 2010 LCMS system with Shimadzu autosampler, Shimadzu binary LC pumps, and Shimadzu PDA detector. The same HPLC column was used for the LC/MS analysis as for the HPLC method. The LC elution conditions were modified for the analysis to the following: Pump A: 0.1% formic acid in water; Pump B: 0.1% formic acid in acetonitrile; time 0 min (10% B), 38 min (30% B), and 60 min (30% B) at a flow rate of 0.3 mL/min. The analysis was done in negative ion mode, with nebulizing gas (nitrogen) flow at 1.5 L/min, collision energy 1.4 kV, mass range 150–600 amu, heat block temperature 200°C, and curved desolvation line heater temperature 250°C.

### Calculations of CGA in the samples

Two methods were utilized to determine the percent by weight of CGA in the samples. The first and most commonly used published method was to use 5-CQA as the standard for all the CGA, which includes the monomers and dimers (single standard method). The second method utilized the ratios of published^14^ extinction coefficients of monomers and dimers at 325 nm (extinction coefficient method). Monomers were calculated based on their ratio compared with 5-CGA = 1.00, 4-CGA 0.92, and 3-CGA 0.94. The very similar extinction coefficients of dimers were averaged and their ratio to 5-CQA was 1.65. The almost identical extinction coefficients of feruloylquinic acids were averaged and their ratio to that of 5-CQA was 0.99.

### Statistical evaluation

Statistics were done using SigmaPlot version 12.5 from Systat Software (Chicago, IL).

## Results

**FIG. 2.**
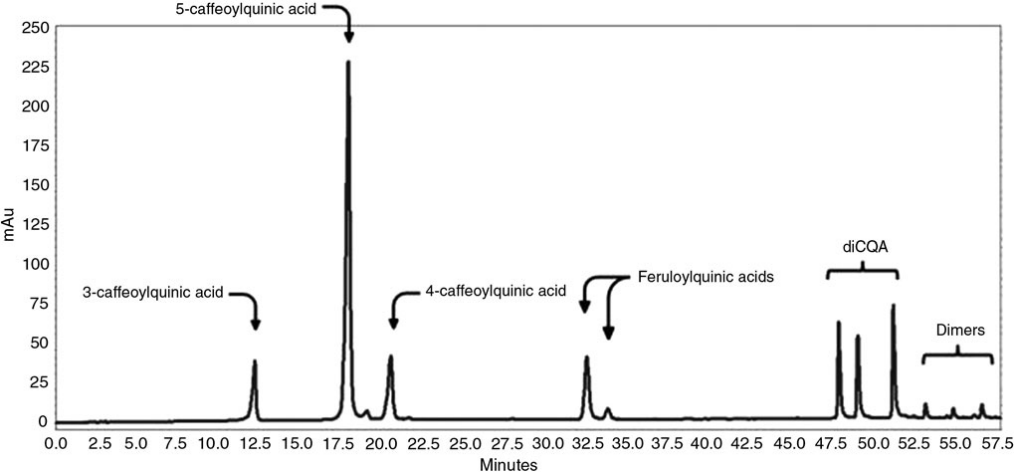
HPLC chromatogram of CGAs in a typical green coffee extract at 325 nm (Sample No. 42). CQA, chloroquinic acid; HPLC, high-pressure liquid chromatography.

**FIG. 3.**
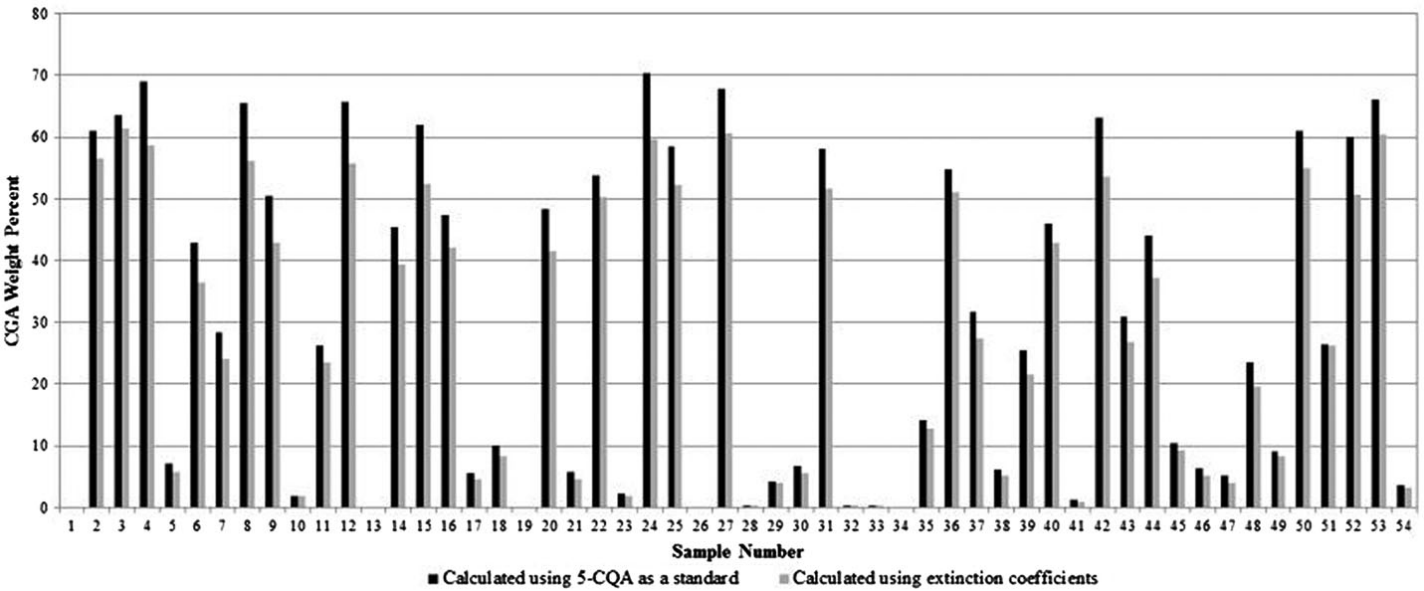
Results of weight percent of total CGAs in green coffee extract samples using two different methods of calculation.

The commercial 5-CQA standards were similar in purity as evaluated by HPLC. Average values were Sigma 96.2%, Chromadex 98.3%, and Phytolab 98.2%. The Sigma product was chosen due to its lower cost. A typical HPLC chromatogram of one of the GCE samples is shown in Figure 2. The retention time of the major CGA 5-CQA was 18 min using the gradient HPLC system. The structures of the monomers and dimers in the samples were confirmed by LC/MS as shown in Table 1. Figure 3 illustrates the results of analysis of the 54 commercial samples using both calculation methods as described previously. The average CGA concentration was 29.9% using 5-CQA as a single standard and 26.8% when using extinction coefficients. A paired *t*-test indicated a very significant difference between the two methods (*P* < .001), and the two methods were significantly correlated with a Pearson correlation coefficient of 0.9978. Both methods were equally precise as determined by statistics; the single standard method averaged 3.65% and the extinction coefficient 5.29% for five different samples of GCE done in quadruplicate ranging from 42% to 62% CGA. We believe the extinction coefficient is the more valid of the two methods as it utilizes data from multiple compounds present in green coffee bean extracts rather than from a single compound. Unfortunately, analytical standards for all the CGA are not commercially available as >99% pure compounds and at a reasonable price.

**Table 1.** Molecular Ions for Peaks Found in Representative Chlorogenic Acid Sample Using Negative Ion Electrospray Ionization Liquid Chromatography/Mass Spectrometry Analysis

*Compound*	*Retention time (minutes)*	*(M–H)^−^ (m/z)*
Chlorogenic acids		
3-Caffeoylquinic acid	20.0	353
5-Caffeoylquinic acid	24.5	353
4-Caffeoylquinic acid	25.5	353
Feruloylquinic acids	26.0	367
	33.0	367
Caffeoylquinic acid dimers	42.3	515
	44.0	515
	46.0	515
Feruloylquinic acid dimers	48.3	529
	49.0	529
	51.7	529
	54.0	529

**FIG. 4.**
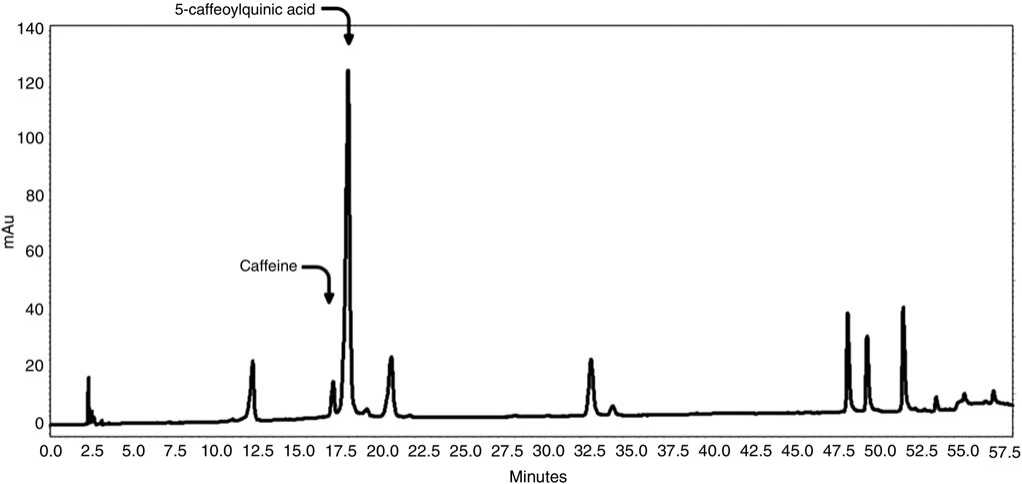
HPLC chromatogram of CGAs and caffeine at 275 nm. Nonattributed peaks are the same as in Figure 2.

**FIG. 5.**
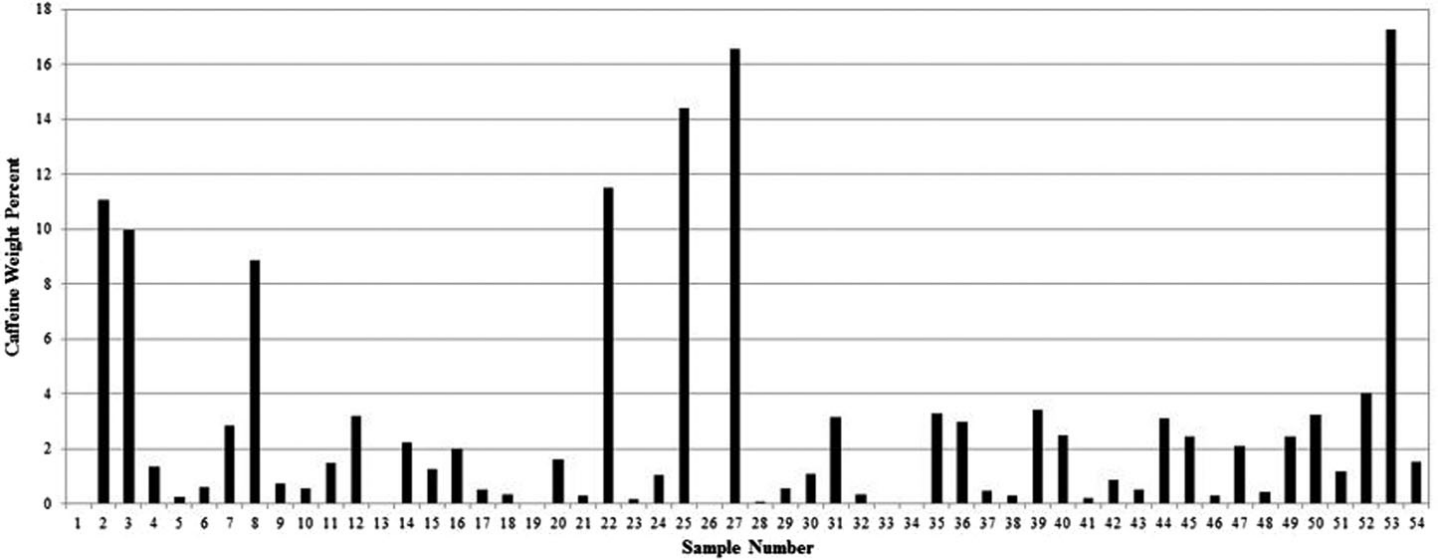
Caffeine weight percent in green coffee extract samples.

Caffeine appeared at 17 min in the HPLC gradient system and could be separated from the CGA when measured at 275 nm (Fig. 4). Results of the caffeine analysis in all the commercial samples are shown in Figure 5. The caffeine averaged 2.8% by weight in the samples. Five of the samples had no caffeine and four of these samples also contained no CGA. Some products were advertised as decaffeinated although they contained detectable levels of caffeine; however, the dose was <2 mg per capsule. The maximum dose of caffeine was a substantial 69 mg/capsule, similar to the amount found in a cup of regular brewed coffee.

## Discussion

The analysis of bioactive compounds found in coffee has been reviewed.^15^ All methods employed chromatography, except for one study that employed UV analysis. The UV results demonstrated that interferences were significantly higher than using HPLC. A recent multilaboratory study used fast HPLC and 5-CQA as a single standard and described an HPLC method requiring a 14-min run time.^13^ The CGA content of our samples varied from 0% to 61.3% as measured by extinction coefficients. In our study, the single standard method gave significantly higher values (average of 5.9% higher) than the extinction coefficient method (*P* < .05 by a paired *t*-test) due to the overestimation of dimeric forms, which have a higher extinction coefficient than monomerics. For the extinction coefficient method, the mean CGA content was 26.8 ± 22.9% and the median was 24.1%.

Of the samples, the two with the most numerous listed ingredients were no. 23 and no. 28, with a total of 10 ingredients. Interestingly, the list was identical in all 10 ingredients and in the order of ingredients, suggesting that one manufacturer may have copied the list of ingredients from the other. Sample no. 23 had almost no CGA (2%) and no. 28 had none. The average commercial capsule weighed 0.57 g, with a range of 0.35–0.89 g for the 54 samples evaluated. Of those samples listing the amount of CGA per dose of GCE on the label (*n* = 39), the average analyzed amount was 157 mg, whereas the labeled amount averaged 233 mg. Thus, the actual amount per dose averaged only 57% of the label, a little over half. A significant number of products (13/54) had <50% of the labeled value upon analysis in our study.

Although the effective dosage of GCE for use as a weight loss supplement has not been definitively established,^4^ the minimum daily dose used in the two earlier published studies^4^ that resulted in significant weight loss was 200 mg of CGA. Based on analyzed CGA content and suggested dose, 31/54 (57%) products would provide at least 200 mg of CGA per day. There were three samples that contained the trademarked product used in the two published clinical studies. Two out of three of these had <10% CGA. The marketing literature touts 50% CGA as being the gold standard of GCE products. Based on this value, only 28% of the commercial products we analyzed met this goal. Thus, it is likely that some of the consumer complaints about the lack of efficacy of GCE may simply be due to the low and variable dose of CGA found in the product they used. It is very difficult to properly assess clinical efficacy of a nutritional supplement when there is no control of the process to make it, thus leading to high variability in product potency. Extraction solvents, time and temperature, and storage stability may all play a role in the high variability in CGA content observed among the formulations evaluated in this study. Stricter regulation of the process to prepare these products will prevent this large variability in commercial supplements. In the meantime, as the adage says, “let the buyer beware.”
